# Antibacterial Drug-Release Polydimethylsiloxane Coating for 3D-Printing Dental Polymer: Surface Alterations and Antimicrobial Effects

**DOI:** 10.3390/ph13100304

**Published:** 2020-10-12

**Authors:** Hang-Nga Mai, Dong Choon Hyun, Ju Hayng Park, Do-Yeon Kim, Sang Min Lee, Du-Hyeong Lee

**Affiliations:** 1 Institute for Translational Research in Dentistry, Kyungpook National University, Daegu 41940, Korea; maihangnga1403@knu.ac.kr; 2 Department of Polymer Science and Engineering, Kyungpook National University, Daegu 41566, Korea; dong.hyun@knu.ac.kr (D.C.H.); pjh99279@naver.com (J.H.P.); 3 Department of Pharmacology, School of Dentistry, Kyungpook National University, Daegu 41940, Korea; dykim82@knu.ac.kr (D.-Y.K.); leeyang2324@naver.com (S.M.L.); 4 Department of Prosthodontics, School of Dentistry, Kyungpook National University, Daegu 41940, Korea

**Keywords:** 3D-printing, dental polymer, antibacterial agent, coating, mesoporous silica nanoparticles, polydimethylsiloxane

## Abstract

Polymers are the most commonly used material for three-dimensional (3D) printing in dentistry; however, the high porosity and water absorptiveness of the material adversely influence biofilm formation on the surface of the 3D-printed dental prostheses. This study evaluated the effects of a newly developed chlorhexidine (CHX)-loaded polydimethylsiloxane (PDMS)-based coating material on the surface microstructure, surface wettability and antibacterial activity of 3D-printing dental polymer. First, mesoporous silica nanoparticles (MSN) were used to encapsulate CHX, and the combination was added to PDMS to synthesize the antibacterial agent-releasing coating substance. Then, a thin coating film was formed on the 3D-printing polymer specimens using oxygen plasma and thermal treatment. The results show that using the coating substance significantly reduced the surface irregularity and increased the hydrophobicity of the specimens. Remarkably, the culture media containing coated specimens had a significantly lower number of bacterial colony formation units than the noncoated specimens, thereby indicating the effective antibacterial activity of the coating.

## 1. Introduction

Three-dimensional (3D) printing technology is an additive manufacturing method in which a 3D object is formed by adding successive layers of material [1,2]. Dentistry has benefited from the rapid expansion of 3D-printing methods, especially in the field of prosthesis manufacturing [3,4,5]. The 3D-printing technology used in dentistry is classified into four main categories: extrusion printing, inkjet printing, laser melting/sintering, and stereolithography printing [3]. Those printing methods are based on the principle of layered manufacturing, which is more suitable than conventional casting and milling methods for producing individualized complex structures [6]. Moreover, using a machining process with computer-aided design/computer-aided manufacturing (CAD/CAM) reduces manual labor and material waste [1]. Recent studies have shown that 3D-printed dental prostheses had a clinically acceptable degree of precision [3,6,7].

Along with the increasing accuracy of 3D-printing technology, various materials, including polymers, ceramics, and metallic powders, have been developed [4]. Regarding dental materials used in 3D printers, polymers are the most commonly used in dentistry for interim and definitive prostheses due to their suitable mechanical strength, highly biocompatible properties, and ease of manipulation [3,4]. However, a major clinical complication associated with dental polymer prostheses is dental plaque surface deposition, which comprises numerous oral microorganisms due to the porosity and water absorptiveness of the polymer material [8]. Specifically, as the 3D-printed prosthesis is generated layer-by-layer, micropores are created when air is trapped between the layers during the printing process or when the individual layers are incompletely fused [9]. During clinical occlusal adjustment and clinical use, these micropores are exposed on the surface because of the abrasion of the restorations, potentially becoming a host for bacterial growth [6]. The risk of material contamination by microorganisms is a critical limitation for the longevity of 3D-printed polymer prostheses [3].

Several strategies have been reported for the fabrication of antimicrobial polymers for 3D-printed polymers [10]. Direct incorporation of an antibacterial agent into dental polymers has been used to reduce plaque accumulation [11]. Chlorhexidine (CHX) is an antiseptic agent widely used in dentistry for its broad-spectrum antibacterial effects and nontoxicity toward mammalian cells [12]. CHX is effective in managing infected oral mucositis; thus, it has commonly been used to prevent dental plaques and to control infections as a topical agent in daily mouth rinse and as an irrigation solution in endodontic treatment [13]. When CHX was directly mixed with the polymer and cured, CHX release at the therapeutic dose was maintained for 28 days [14,15,16]. However, direct mixing may negatively affect the mechanical and surface properties of polymers in terms of polymerization degree, surface porosity, and water absorption [11,17]. Moreover, commercial polymers used for the subtractive manufacturing method are provided in a completely polymerized state, making it difficult to directly add CHX inside the substance without jeopardizing material integrity [18].

An alternative technique for inhibiting bacterial adherence is to change the surface property of the object by creating a coating film [19,20,21]. Coating layer formation does not sacrifice the mechanical properties and integrity of the material. The major mechanisms underlying the antibacterial activity of the coating layers include antiadhesion/bacterial-repelling and contact-killing [22]. Antiadhesion coating reduces the adhesion force between bacteria and the substrate to enable the easy removal of bacteria before the biofilm layer is formed [21]. Alternatively, in the contact-killing approach, antibacterial compounds are attached to the surface of the material by flexible, hydrophobic polymeric chains, which can kill bacteria upon contact [21,22]. Remarkably, using a polymer coating significantly reduced plaque biofilm formation on polymeric restorations with the coating layer exhibiting acceptable mechanical and chemical durability [20]. Azuma et al. [19] reported that silica coating with silica nanoparticles of various sizes was effective in decreasing bacterial adherence to polymeric restorations. While these previous coating materials showed antiadhesion potential, they passively hindered the adherence of early colonizers by increasing polymer surface hydrophobicity [19,20]. This strategy may physically suppress dental plaque formation and maturation; however, it provides no active antimicrobial agent to inhibit the vitality and growth of pathogenic microorganisms.

With the development of new drug-carrier materials, active antibacterial agent-releasing coating is now a significant focus in biomedical research [22]. In its most advanced form, the coating mediates antibacterial activity by releasing loaded antibacterial compounds over time to kill the bacteria in the surrounding [21,22]. In drug delivery systems, materials with a porous structure are recommended for incorporating drug-loaded particles [23,24]. Among the porous polymeric materials, polydimethylsiloxane (PDMS) has been used in the medical field due to its flexibility, biocompatibility, transparency, low cost, and ease of fabrication [23,25]. The incompatibility between the coated substrate and PDMS could cause a dewetting that may induce a greater surface roughness; however, the subsequent treatments of oxygen plasma could enhance the wettability of precured PDMS, inducing its uniform coating [26,27]. Moreover, the drug release rate of PDMS can be controlled by surface modifications using plasma treatment, which effectively influence the water penetration rate and functionalization of the polymer that contains drug [28]. The highly functionalized PDMS can be polymerized using thermal treatment or ultraviolet (UV) light activation for curing the coating layer, inducing a more chemically and physically stable coating layer on the substrate [27]. Thus, it would be an effective approach for tissue engineering or drug delivery systems in biological and biomedical applications [29]. In dentistry, PDMS coating has been used for tooth enamel and metallic dental implants as a hydrophobic layer to improve the antibacterial features [30]. However, no studies have examined the PDMS coating application for active antibacterial effects in dental 3D-printable polymers. Therefore, this study evaluated the effect of a newly developed CHX-loaded PDMS-based coating on the surface microstructure, surface wettability, and antibacterial activity of the 3D-printing dental polymer.

## 2. Results

### 2.1. Surface Characteristics

**Figure 1:**
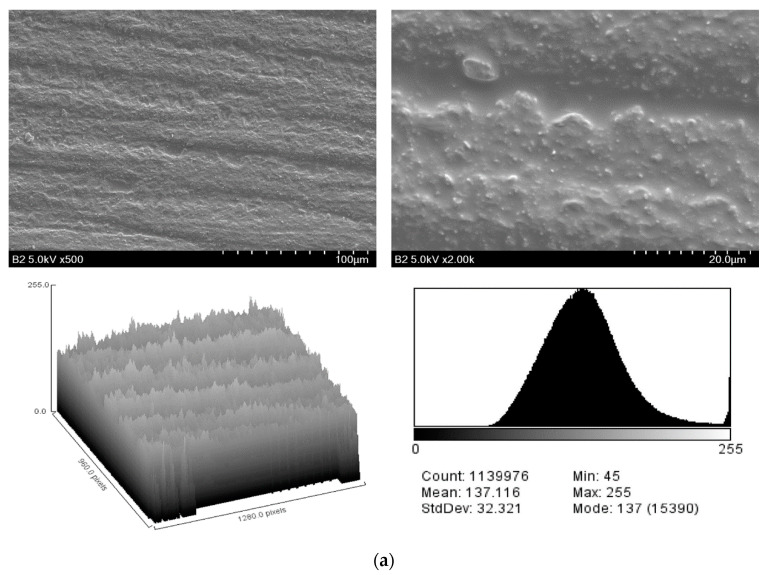

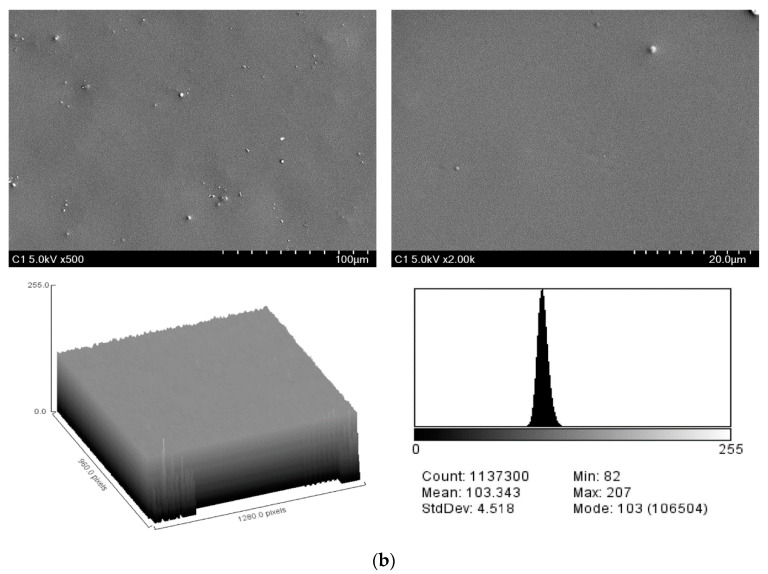
Scanning electron microscope (SEM) images at 500 × (left) and 2000 × (right) magnification and the resulting histogram of pixel brightness levels (0 = black, 255 = white) of: (**a**) noncoated specimen; and (**b**) coated specimens.

Figure 1 shows the microscopic images of specimens in different groups. Deep grooves and small defects were found on the surface of noncoated specimens, whereas no scratches were observed on the surface of coated specimens. Several different-sized silica particles were observed on the coating surface.

**Figure 2:**
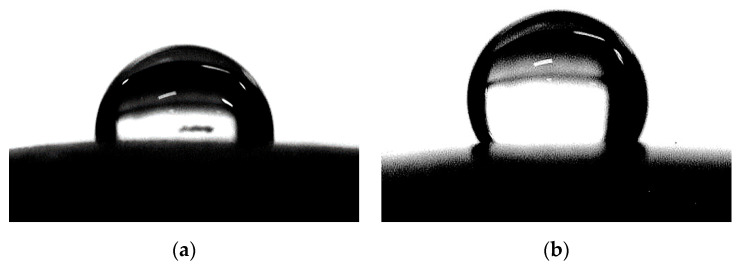
Contact angle of the specimens: (**a**) noncoated specimen; and (**b**) coated specimen.

Table 1 presents the SEM image analysis for surface roughness obtained from the surface plot histograms. The SEM roughness index (SRI) values were significantly lower in the coated group than in the noncoated group (*p* < 0.001). Regarding surface wettability, the mean contact angles were significantly higher in the coated group than in the noncoated group (*p* < 0.001) (Figure 2 and Table 1).

### 2.2. Antimicrobial Activity

**Figure 3:**
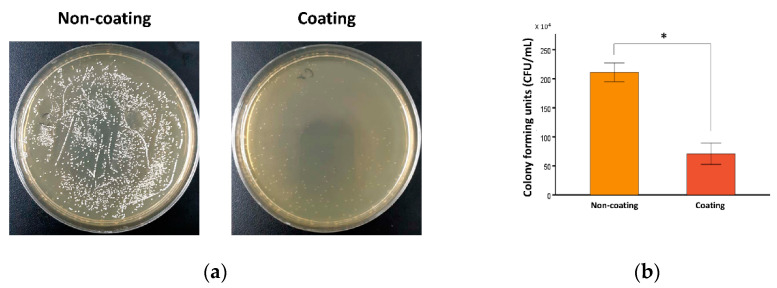
Bacterial growth inhibition assay of noncoated and coated groups: (**a**) bacterial colonization on agar plates; and (**b**) colony-forming units. Statistical analysis was performed using three independent technical replicates, inducing similar biological results. Independent *t*-test showed that the culture media containing the coated specimens had significantly lower CFU values than the culture media containing the noncoated specimens (* *p* < 0.001).

Because the specimen itself could affect bacterial growth, the incubation of *S. mutans* with noncoated specimen was a control in this experiment. Statistical analysis was performed using three technical replicates. Biological relevance of the results was confirmed using three independent experiments with similar results. Figure 3 shows the results of the *S. mutans* bacterial growth inhibition assay for the noncoated and coated groups. The numbers of bacterial colonies (×10^4^ CFU/mL) of the noncoated and coated groups were 210.92 ± 8.02 and 70.76 ± 9.16, respectively. Independent *t*-test showed that the culture media containing the coated specimens had significantly lower CFU values than the culture media containing the noncoated specimens (*p* < 0.001).

## 3. Discussions

This study evaluated the effect of a newly developed CHX-loaded PDMS-based coating substance on the surface properties and antibacterial ability of coated 3D-printing dental material. From the results of this study, applying the coating layer on the polymer specimens significantly reduced their surface irregularity while increasing the hydrophobicity and antibacterial activity.

Once a dental restoration is placed in the oral cavity, proteins from saliva cover the surface of the restoration as a film, followed by the attachment of free-floating bacteria to the film by microfilaments in the cell walls to form a biofilm on the restoration [31]. Restorations with greater surface roughness show higher biofilm formation because the irregular surface geometry offsets shear forces to the surface, thereby providing a favorable context for bacterial growth [32]. In the clinical context, surface smoothing of dental restoration by using polishing tools is necessary before restoration is placed in the mouth [33]. The microscopic images of this study showed sharp grooves and defects on the surface of specimens in the noncoated group due to the grinding motion during the polishing process. The result agrees with previous studies where the polishing tools caused some microdefects to the restoration surface as they removed material by abrasion [34]. In specimens given the coating substance, scratches and defects were unobserved, and the roughness value was significantly lower than that of the noncoated specimens. The results suggest that the coating decreases surface irregularity by filling the scratches resulting from polishing and the inherent micropores created by the 3D-printing process. Previous studies that evaluated the effect of different surface treatments on dental ceramic restoration also indicated a significant decrease in roughness when a thin layer of glazing material was coated on the restoration surface [35]. In addition, various micrometer-sized particles were observed on the surface of the coated specimens. Considering that the mesoporous silica nanoparticles (MSNs) dispersed in the solvent were nanosized, this result implies that some of the silica particles on the coated layer became aggregated and clustered. A silica coating layer needs to contain particles of an optimum size to reduce microorganism adherence [19,36]. Thus, further technical optimization is needed to improve the homogeneous distribution of the silica nanoparticles and to minimize their uncontrolled aggregation in the coating layer.

Surface free energy greatly influences the initial step of biofilm formation [37]. Lower surface energy of the material has weakened bacterial adhesion; thus, the bacteria that adhere to a material with low surface energy are more easily removed by an external force [37,38]. The work of adhesion can be calculated by measuring the contact angle of the liquid to the solid surface [39]. For a low wetting surface, the surface energy of the solid is weaker than the surface tension of the liquid, allowing the liquid to easily retain its droplet shape. Therefore, a higher contact angle is related to lower surface energy and low interfacial tension of the solid surface. In this study, the coated group showed a significantly higher contact angle than the noncoated group, thereby indicating a lower surface energy of the coated specimens than that of the noncoated specimens. This is due to the hydrophobicity and low surface energy of PDMS, which is a beneficial feature of the material that contributes to the low bacterial adhesion [40].

*S. mutans* is an important etiologic agent for initiating dental caries [41]. The acid produced from this bacteria decays tooth structure and induces restorative treatment failure [41]. Accordingly, dental restorations require antibacterial qualities to ensure long-term success. For this purpose, the MSN was used to encapsulate CHX in this study, and the CHX@MSN was then combined with PDMS to unprecedentedly synthesize an antibacterial coating substance for polymeric restorative 3D-printing dental material. The bacterial growth inhibition assay results showed that the coated specimens had antimicrobial activity against *S. mutans.* The antimicrobial activity may be due to CHX release from the coating substance. Adding CHX to dental restorative material could increase its antibacterial activity [14,15]. Yan et al. [12] incorporated CHX@MSN into a glass ionomer cement powder and showed that CHX was continuously released, and the antibiofilm effect was maintained up to 30 days. Remarkably, dental resin composites with CHX@MSN showed controlled release of CHX over a prolonged time, providing strong inhibition against *S. mutans* adherence [11]. However, the antibacterial activity of previous coatings has been limited to the surface of the coated objects. In our study, an elastic porous material, PDMS, was used to store and release the CHX@MSN particles. The encapsulation and drug-loading efficiency of the CHX@MSN were recorded at rates of 25.22% and 63.04%, with a stable CHX releasing rate of approximately 1.56 µg/mL within the first 24 h in a pilot study [32]. Because the synthesized coating layer could release the loaded CHX over time [42], the coating exhibited antibacterial effects in the surrounding areas not directly in contact with the surface of the restoration. Moreover, the CHX@MSN and PDMS materials used in this study have been reported to be relatively noncytotoxic [32]. Therefore, this active antibacterial protective film formation is expected to be a novel method for actively inhibiting bacterial inhabitation around the coated surface of restorations.

Note that the human oral environment is more complicated than the in vitro experimental context because the oral cavity temperature, food intake, and the pH and composition of saliva vary between subjects and even within a subject [43]. In addition, the mechanical properties and material stability of the coating layer were not investigated in this study. However, the mechanical properties of PDMS strongly depend on the mixing ratio of a base to a curing agent in PDMS mixture [26,27,44]. In this study, the PDMS mixture comprises a base and a curing agent at a weight ratio of 5:1 with the relative elastic modulus number of 3.59 MPa [44]. However, the mechanical properties of the coating can be significantly improved by incorporating MSN into the PDMS as the elastic modulus of MSN is at least four orders of magnitude larger than that of PDMS [45]. Moreover, the hydrogen bonds between MSNs and covalent bonds between MSN and PDMS may induce a strong resistance to mechanical deformation [46]. The findings in those previous studies indicates that the mechanical strength of the PDMS coating layer can be tuned by controlling the input parameters such as the curing agent and MSN. In this study, the surface microstructure, surface wettability, and antibacterial activity were immediately evaluated after coating and within 24 h of drug releasing; thus, the material was expected to be stable during this stage. A further study that focuses on investigating the mechanical properties of the coating should be conducted in clinically relevant conditions to extend the understanding of this new material.

## 4. Materials and Methods

### 4.1. Synthesis of the *Coating Material* (CHX@MSN-Loaded PDMS)

**Figure 4:**
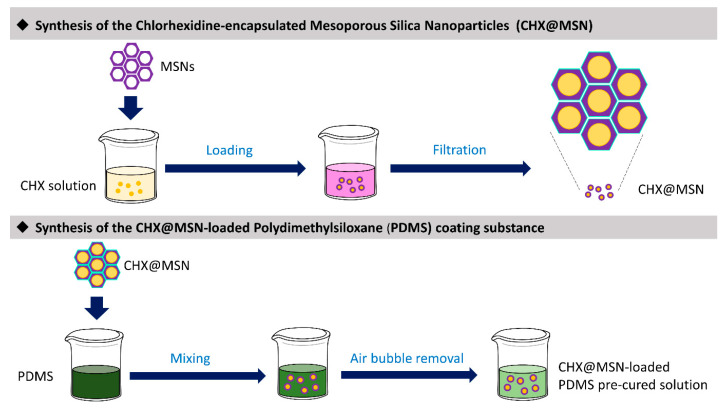
Synthesis of the CHX@MSN-loaded PDMS coating substance. CHX, Chlorhexidine; MSN, Mesoporous silica nanoparticles; CHX@MSN, Chlorhexidine encapsulated in mesoporous silica nanoparticles; PDMS, Polydimethylsiloxane.

Figure 4 illustrates the fabrication process for the coating substance. Fifty milligrams of CHX (Sigma-Aldrich Co., St. Louis, MO, USA) were dissolved in 5 mL of absolute ethanol, and then dried MCM-41 mesoporous silica nanoparticles (MSN) (Sigma-Aldrich Co., St. Louis, MO, USA) with a pore volume of 0.98 cm^3^/g, pore size of approximately 2.5 nm, and Brunauer–Emmett–Teller (BET) surface area below 1000 m^2^/g were dispersed into the CHX solution. The mixture was sonicated for 10 min and incubated for 24 h at room temperature using a magnetic stirrer (Corning PC-420D, Fisher Scientific, Lowell, MA, USA) at a speed of 300 rpm. Next, to collect the CHX@MSN particles, the mixture was filtered (Labogene Scan Speed Mini, Lynge, Denmark) and then vacuum-dried (OV-11, JEIO Tech, Seoul, Korea).

The CHX@MSN particles were then mixed with PDMS (Sylgard 184, Dow Corning, Midland, MI, USA) solution at 0.4 wt% relative to the total PMDS mass. The PDMS mixture comprised a base and a curing agent at a weight ratio of 5:1. All components were blended in a SpeedMixerTM (FlackTek Inc., Landrum, SC, USA) for 3 × 30 s to form a precured coating paste, which was stored in the dark at room temperature and vacuumed for 20 min to remove bubbles before use.

### 4.2. Coating Procedure

Sixty disk-shape specimens (*N* = 60) with a thickness of 1.0 mm and diameter of 13.0 mm were designed using CAD software (Geomagic Design X, 3D Systems, Inc., Rock Hill, SC, USA) and printed with the photopolymer (RAYDent C&B, Ray Co., Hwaseong, Korea) using a digital light processing 3D printer (RAM500, Ray Co., Hwaseong, Korea). All specimens were postcured with a curing unit (RPC500, Ray Co., Hwaseong, Korea) for 20 min and polished with a 1000-grit silicon carbide abrasive paper (Buehler GmbH, Dusseldorf, Germany) for 60 s. Table 2 provides the composition of the photopolymer.

**Figure 5:**
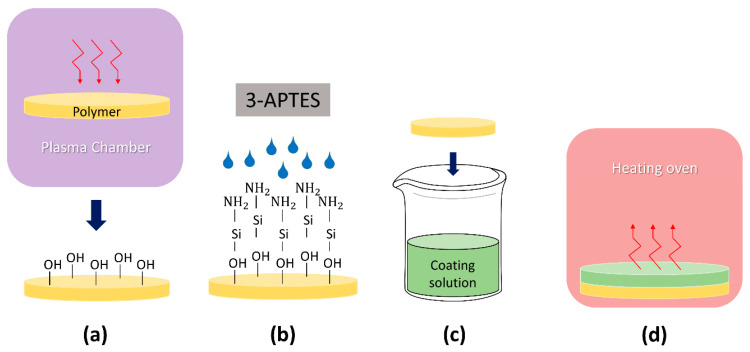
Surface functionalization and coating treatment: (**a**) oxygen plasma treatment; (**b**) APTES treatment; (**c**) dip coating; and (**d**) Thermal curing. APTES, 3-aminpropyltriethoxysilane; PMMA, polymethyl methacrylate; CHX@MSN, Chlorhexidine encapsulated in mesoporous silica nanoparticles; PDMS, Polydimethylsiloxane.

Surface functionalization and coating process were performed in the coated group specimens (*n* = 30). The polished specimens were cleaned with isopropyl alcohol and treated with oxygen plasma (CUTE, Femto Science Co., Seoul, Korea) for 5 min (Figure 5a). The specimen surfaces were functionalized by immersion into 5% (*v*/*v*) 3-aminopropyltriethoxysilane (APTES) (Sigma-Aldrich Co., St. Louis, MO, USA) solution at 85 °C for 10 min (Figure 5b). The specimens were then coated by dipping in the precured coating solution using a dip coating equipment (KD Scientific, Holliston, MA, USA) at a lowering speed of 6000 µm/s and lifting speed of 1000 µm/s (Figure 5c). Subsequently, the specimens underwent thermal treatment in an oven (OV-11, JEIO Tech, Seoul, Korea) at 80 °C for 2 h (Figure 5d). The noncoated group specimens were defined as control (*n* = 30).

### 4.3. Evaluation of Surface Microstructure

Surface microstructure was evaluated using a scanning electron microscope (SEM) (S-4500, Hitachi Co., Tokyo, Japan). Specimens were coated with spotted platinum using a sputter coater (E-1030, Hitachi Co., Tokyo, Japan), and scanned at 5 kV at 500× and 2000× magnification. Ten locations in each specimen were randomly selected for image acquisition. The images were converted to 8-bit grayscale (black = 0, white = 225) using ImageJ software (version 1.52k, National Institute of Health, Bethesda, MD, USA). The pixel brightness data of each image were plotted as 256-level histograms to build a surface plot image, where the y-axis represents the 0–255 grayscale levels and the x-axis represents the pixel frequency. The histograms from all 10 images were combined, and the standard deviation of the pixel brightness was calculated as the SRI [47].

### 4.4. Measurement of Surface Wettability

**Figure 6:**
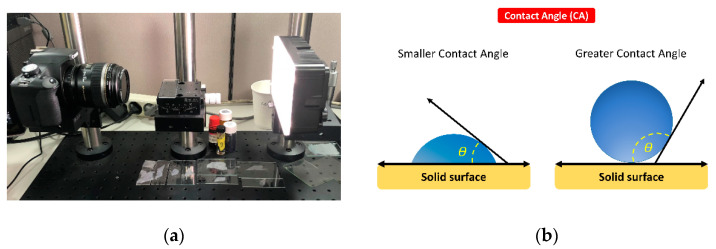
Measurement of surface wettability: (**a**) contact angle measurement system setting; and (**b**) contact angle (*θ*) measurement by the tangent angle of the liquid droplet to the surface.

Surface wettability was evaluated by measuring the contact angle (CA) in an air environment. A liquid droplet (5 µL) of distilled water was dispensed onto the specimen surface at a room temperature of 20 °C. The image of the droplet was immediately captured by a digital camera (Canon EOS 500D with Canon EF 100 mm f2.8 Macro USM Lens, Canon Inc., Tokyo, Japan) (Figure 6a), and the contact angles were determined from the corresponding pictures using the contact angle plugin of the ImageJ software. The CA degree was determined by measuring the tangent angle to the surface of the liquid droplet (Figure 6b). The mean value was calculated by averaging three individual measurements.

### 4.5. Assessment of Antimicrobial Activities

To evaluate the antibacterial activity, a bacterial growth inhibition assay was performed. *Streptococcus mutans* ATCC 25175 (*S. mutans*) was inoculated into a 15-mL tube containing 10 mL of brain heart infusion (BHI) media (BHI, Mast Group, Bootle, UK) and incubated on a rotary shaker (150 rpm) at 37 °C. To determine the concentration of bacteria in culture media, the optical density at 600 nm (OD_600_) of the cell suspensions was recorded using a DS-11+ apparatus (DeNovix, Wilmington, DE, USA). After 12 h, OD_600_ was checked to measure bacterial density in liquid media.

When the OD_600_ of the *S. mutans* culture reached approximately 0.4, aliquots of 500 μL were added to each well in a 24-well plate and incubated with noncoated or coated specimens at 37 °C on a shaker at 15 rpm for 24 h. Cultures without any test specimens were used as the control condition. Following incubation, appropriate dilutions of the cultures were plated on BHI agar plates using an ethanol-flamed bacterial spreader, and the plate was incubated for 24 h at 37 °C. Then, the colony-forming units per milliliter (CFU/mL) were calculated using the number of colonies observed and the dilution factor (1:10^4^) for each well.

### 4.6. Statistical Analysis

Statistical calculations were performed using SPSS software (SPSS version 25.0, IBM Inc., Chicago, IL, USA). The measured values were expressed as the mean ± standard deviation. The independent *t*-test was conducted to compare differences between the groups. The statistical significance level was set at 0.05.

## 5. Conclusions

This study evaluated the effect of a newly developed CHX@MSN-loaded PDMS-based coating substance on the surface properties and antibacterial ability of coated 3D-printing dental material. From our results, it can be concluded that applying the coating layer on the polymer specimens significantly reduced their surface irregularity, while increasing the hydrophobicity and antibacterial activity.

## Figures and Tables

**Table 1:** Effect of coating layer on SEM roughness index (SRI) and contact angle degree (CA) measurements.

Group	Mean (SD)		
	SRI	CA	*p*-Value
**Noncoated**	39.25 (3.69) ^a^	120.22 (4.46) ^a^	<0.001
**Coated**	15.41 (8.07) ^b^	91.88 (8.19) ^b^	

^a, b^ Different superscript lowercase letters indicate a statistically significant difference within a column; SRI, SEM roughness index; CA, contact angle degree.

**Table 2:** Composition of the 3D-printing photopolymer.

Component *	Content (%)
α,α’-[(1-Methylethylidene)di-4,1-phenylene] bis [ω-[(2-methyl-1-oxo-2-propenyl) oxy] poly (oxy-1,2-ethanediyl)	20–35
7,7,9(or 7,9,9)-Trimethyl-4,13-dioxo-3,14-dioxa-5,12-diazahexadecane1,16-diyl 2-methyl-2-propenoate	20–28
2-Methyl-2-propenoic acid 1,2-ethanediylbis(oxy-2,1-ethanediyl) ester	20–25
Phenylbis (2,4,6-trimethylbenzoyl) phosphine oxide	1–10
Rutile (TiO2)	0.1–5

* As provided by the manufacturer.
